# Improvements in Oral Lichen Planus Following Periodontal Treatment in a Patient With Metabolic Dysfunction-Associated Steatotic Liver Disease: A Case Report

**DOI:** 10.7759/cureus.65054

**Published:** 2024-07-21

**Authors:** Yumiko Nagao, Hitomi Nakagaki, Nobuo Tomiyasu, Masahide Tsuji

**Affiliations:** 1 Public Health, Graduate School of Medicine, Juntendo University Faculty of Medicine, Tokyo, JPN; 2 Dental and Oral Surgery, Tsuji Dental and Oral Surgery Clinic, Omuta, JPN; 3 Internal Medicine and Gastroenterology, Kohama Clinic, Omuta, JPN

**Keywords:** medical-dental collaboration, hepatic dysfunction, metabolic dysfunction-associated steatotic liver disease, periodontitis, oral lichen planus

## Abstract

Oral lichen planus (OLP), a chronic inflammatory mucocutaneous disease, is known to be associated with liver disease. Additionally, associations between periodontal disease and metabolic dysfunction-associated steatotic liver disease (MASLD), as well as cardiovascular disease, have been reported. Herein, we report a case of a 68-year-old male who presented at a dental clinic with OLP, which showed signs of improvement after treatment for periodontal disease. The patient had hepatic dysfunction and steatosis, which was complicated by angina pectoris. He was diagnosed with OLP and periodontal disease. Subsequent close examination of his liver led to a diagnosis of MASLD. Treatment for periodontal disease and enhanced oral self-care improved the OLP lesions and liver function values. This case demonstrates that collaboration between different medical disciplines can significantly impact patient health.

## Introduction

In recent years, the prevalence of metabolic dysfunction-associated steatotic liver disease (MASLD, formerly known as nonalcoholic fatty liver disease (NAFLD)) has rapidly increased worldwide, and it is one of the leading causes of liver disease [1]. MASLD is a hepatic manifestation of the metabolic syndrome, ranging from simple steatosis to an active inflammatory form of metabolic dysfunction-associated steatohepatitis (MASH), which can progress to cirrhosis and hepatocellular carcinoma [2,3]. The prevalence of MASLD is strongly associated with features of the metabolic syndrome, including obesity and type 2 diabetes [1,3].

Recently, the association between MASLD and periodontitis has received considerable attention, with a significantly higher prevalence of the periodontopathogen *Porphyromonas gingivalis* reported in patients with MASLD compared to that in healthy individuals [4]. Evidence suggests that periodontitis may alter the gut microbiota and exacerbate the progression of MASLD [5]. Additionally, periodontal therapy improves liver function parameters in patients with MASLD and decreases HbA1c in diabetic patients [6,7].

Oral lichen planus (OLP) is an inflammatory disease on the oral mucosa closely related to liver diseases, especially hepatitis C [8]. However, studies on OLP and MASLD/MASH are limited [9], and the association between these two conditions remains poorly understood. Herein, we report the case of a 68-year-old male with OLP who presented with improvements in the condition along with mild improvements in liver function values following treatment for periodontal disease. The findings of this study emphasize the importance of close collaborations between dental and medical clinics in the overall management of patients with MASLD and OLP.

## Case presentation

**Figure 1:**
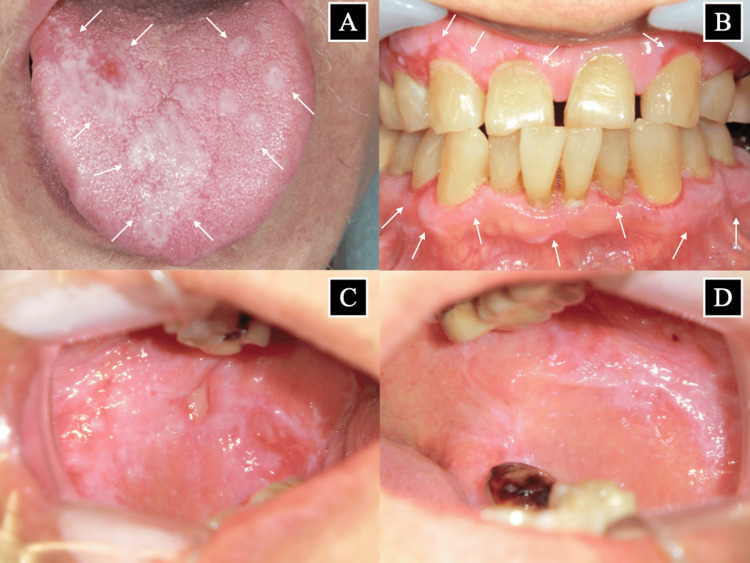
Images of the oral mucosa at the first visit (November 17, 2022) (A) Tongue, (B) gingiva, (C) right buccal mucosa, and (D) left buccal mucosa. White arrows indicate lacy white striated lesions and erosions on the mucosa

A 68-year-old Japanese male visited the Tsuji Dental and Oral Surgery Clinic on November 17, 2022, complaining of pain throughout the oral mucosa. White lesions were found on the buccal mucosa (bilateral), palatal mucosa, tongue, upper and lower gingiva, and lower lip, with erosions on the buccal mucosa, palatal mucosa, and upper and lower gingiva (Figure 1).

**Figure 2:**
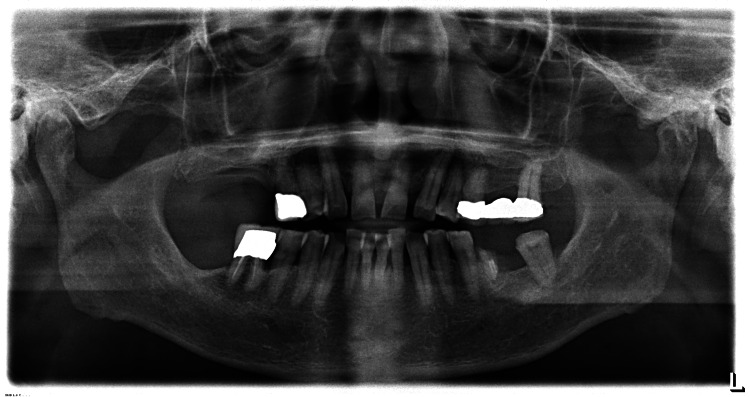
Panoramic radiograph showing generalized moderate to severe periodontitis A radiograph taken in November 2022 demonstrating generalized horizontal alveolar bone loss. The alveolar bone crest is positioned approximately 3-4 mm apical to the cementoenamel junction in most areas, indicating moderate to severe bone loss. These findings are consistent with the clinical diagnosis of generalized moderate to severe periodontitis

The lesions were clinically diagnosed as OLP by an oral surgeon on the characteristic appearance and distribution of the lesions. A biopsy was not performed due to the typical clinical presentation of OLP and the patient's medical history of coronary artery stenting, which required antiplatelet therapy, potentially increasing the risk of bleeding complications from an invasive procedure. Inflammation of the gingiva and horizontal resorption of the jawbone on radiographic examination (Figure 2) were observed, leading to the diagnosis of periodontitis.

Periodontal examination revealed the deepest pockets (5 mm) in #14, #23, #24, #25, #27, and #37. Moderate pockets (4 mm) were found in #15, #36, #35, #34, #33, #32, #31, #41, #42, #43, #44, #45, and #46, while shallower pockets (3 mm) were observed in #13, #11, and #21. In total, 22 teeth remained. These findings indicate generalized moderate to severe periodontitis, consistent with the radiographic observations. The patient did not have a family dentist and was not treated for periodontitis. He was not in the habit of brushing his teeth after each meal.

The patient was 50 years old when a physical examination at work (including blood tests and abdominal ultrasound) indicated hepatic disorder and hepatic steatosis. The patient had previously received advice from an occupational physician to engage in exercise and dietary therapy for weight management but had not adhered to these recommendations. Exercise therapy suggested by an occupational physician did not lead to any improvement in the condition. He had no drinking or smoking habits. He underwent percutaneous coronary stenting at age 63 for angina pectoris and was taking antiplatelet agents (aspirin) and long-term calcium channel blockers (diltiazem hydrochloride).

When the patient was first seen at our hospital on November 17, 2022, he was prescribed beclomethasone dipropionate (50 µg) for pain relief and instructed to spray the medication on the oral mucosa twice a day. He was then referred to a gastroenterologist for a thorough evaluation. Table 1 shows the results of the blood and biochemical tests.

**Table 1:** Summary of the laboratory data a: Workplace medical examinations; b: Only BMI measured on this date; N/A: not available; BMI: body mass index; AST: aspartate aminotransferase; ALT: alanine aminotransferase; LDH: lactic dehydrogenase; ALP: alkaline phosphatase; gamma GTP: gamma glutamyl transpeptidase; HDL: high-density lipoprotein; LDL: low-density lipoprotein; BUN: blood urea nitrogen; CK: creatine kinase; Na: sodium; Cl: chloride; K: potassium; FBS: fasting blood sugar; HBsAg: hepatitis B surface antigen; anti-HBs: antibody to HBsAg; HCV: hepatitis C virus; AFP: alpha fetoprotein; PIVKA-II: protein induced by vitamin K absence or antagonist-II; CA19-9: carbohydrate antigen 19-9; CEA: carcinoembryonic antigen; PSA: prostate-specific antigen; TSH: thyroid-stimulating hormone; FT3: free triiodothyronine 3; FT4: free thyroxine 4; TPHA: Treponema pallidum hemagglutination assay

	Normal range	April 5, 2022^a^	Nov. 17, 2022^ b^	Nov. 30, 2022^a^	Aug. 8, 2023	Nov. 17, 2023
BMI, kg/m^2^	18.5-24.9	N/A	25.3	N/A	N/A	22.3
AST, U/L	13-33	42		33	24	27
ALT, U/L	6-30	49		44	34	40
LDH, U/L	119-229	140		163	138	139
ALP, U/L	38-113	134		137	119	N/A
Gamma GTP, U/L	10-47	36		45	37	41
Total bilirubin, mg/dL	0.3-1.2	0.6		0.8	0.5	N/A
Total cholesterol, mg/dL	128-219	172		176	151	N/A
HDL, mg/dL	40-96	47		43	42	41
LDL, mg/dL	65-139	112		114	99	112
Triglyceride, mg/dL	30-149	88		66	63	86
Total protein, g/dL	6.7-8.3	6.7		7.7	6.8	7.2
Albumin, g/dL	4.0-5.0	4.0		4.6	4.0	4.3
BUN, mg/dL	8.0-22.0	11.1		12.4	12.9	15.4
Creatinine, mg/dL	0.61-1.04	0.81		0.79	0.81	0.90
CK, U/L	62-287	N/A		N/A	46	N/A
Na, nmol/L	138-146	144		141	142	140
Cl, nmol/L	99-109	106		103	105	102
K, nmol/L	3.6-4.9	4.6		4.1	4.8	4.4
FBS, mg/dL	69-109	94		90	87	84
HbA1c, %	4.6-6.2	5.8		5.6	5.5	5.6
CRP, mg/dL	≤0.30	0.04		0.06	N/A	N/A
Qualitative CRP	Negative	Negative		Negative	N/A	N/A
White blood cell, /μL	3500-9800	5400		5800	5500	7300
Red blood cell, 10^4^/µL	427-570	510		546	529	560
Hemoglobin, g/dL	13.5-17.6	14.7		15.3	13.8	14.8
Hematocrit, %	39.8-51.8	47.2		46.9	44.0	46.0
Platelet count, 10^3^/µL	13.0-36.9	21.3		21.9	20.4	23.4
HBsAg	Negative	Negative		Negative	N/A	N/A
Anti-HBs	Negative	Negative		Negative	N/A	N/A
Anti-HCV	Negative	Negative		Negative	N/A	N/A
AFP, ng/mL	≤10.0	2.6		2.5	N/A	N/A
PIVKA-II, mAU/mL	<40	29		33	N/A	N/A
CA19-9, U/mL	≤37	<5		<5	N/A	N/A
CEA, ng/mL	≤3.5	1.5		1.6	N/A	N/A
PSA, ng/mL	≤4.000	3.29		1.98	N/A	N/A
TSH, µIU/mL	0.50-5.00	0.94		0.98	N/A	N/A
FT3, pg/mL	2.30-4.00	3.62		3.83	N/A	N/A
FT4, ng/dL	0.90-1.70	1.29		1.59	N/A	N/A
Qualitative TPHA	Negative	Negative		Negative	N/A	N/A

The body mass index (BMI) at the initial visit was 25.3, indicating obesity. The patient was not infected with hepatitis B or C virus but had abnormal liver function values and hepatic steatosis on abdominal ultrasonography, which confirmed a diagnosis of MASLD (simple steatosis). The patient was informed that OLP is a potentially malignant disease often linked with liver disease. The connection between periodontal disease and MASLD was also explained to him. Furthermore, he was made aware that oral self-care was essential and that treatment of periodontal disease would likely contribute to his overall health.

**Figure 3:**
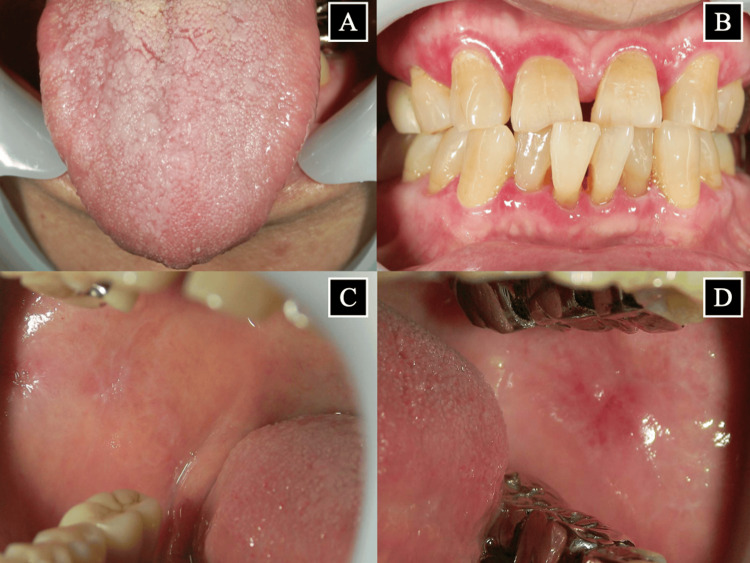
Images of the oral mucosa approximately one year after the start of periodontal treatment (October 6, 2023) (A) Tongue, (B) gingiva, (C) right buccal mucosa, and (D) left buccal mucosa. Lacelike white striated lesions and erosions of OLP have improved, and the pain has disappeared

Oral hygiene instructions were initiated for the patient on November 2022, along with plaque control, scaling, and professional mechanical tooth cleaning. Self-care via tooth brushing and interdental brushing was started after each meal. Periodontal treatment began on November 26, 2022. The initial intensive phase of treatment showed significant improvements within the first two months, with ongoing maintenance and monitoring continuing thereafter. The patient continues to attend regular maintenance visits to ensure periodontal tissue health and to monitor the status of OLP lesions. The average depth of the periodontal pockets in the 22 remaining teeth in the oral cavity was 4.1 mm, and the maximum depth was 5 mm (November 17, 2022) before treatment; the corresponding values improved to 1.9 mm and 3 mm, respectively, after the initial phase of treatment (January 20,2023). Notably, no teeth showed periodontal pockets of 4 mm or deeper posttreatment. Only one tooth (#36) retained a 3 mm pocket, while the majority showed improvements to 2 mm (#15, #23, #24, #25, #27, #36, #35, #34, #33, #32, #31, #41, #42, #43, #44, #45, #46), and four teeth improved to 1 mm depths (#14, #13, #11, #21). Subsequently, signs of improvement were seen in both OLP and periodontitis, along with pain reduction (Figure 3).

The aspartate aminotransferase (AST; 42 U/L) and alanine aminotransferase (ALT; 49 U/L) levels before periodontal treatment decreased to 24-27 U/L and 34-40 U/L, respectively, after treatment, indicating a mild but improving trend in liver function values (Table 1). No change in fat deposits was observed in the abdominal ultrasound. The BMI decreased from 25.3 in November 2022 to 22.3 by November 2023, despite the absence of diet or exercise therapy.

## Discussion

This is the first report of a case in which periodontal therapy improved the OLP lesions and liver function values. The patient had angina pectoris, a cardiac disease that is frequently associated with MASLD [10] and periodontal disease, which is also a complication of cardiac disease [11]. There is a strong possibility of an association between the four diseases (periodontal disease, OLP, MASLD, and cardiac disease). The prevalence of MASLD in Japan is reported to be 25.51% and is expected to affect half of the population by 2040 [12]. Lifestyle modifications are important in treating MASLD and MASH; a healthy diet and exercise are recommended, especially for those with obesity and metabolic syndrome [13].

Tooth brushing behavior and frequency are associated with obesity, and infrequent tooth brushing is associated with both obesity and diabetes [14]. In the current case report, the patient was not on a diet or exercise regimen; however, efficient toothbrushing and treatment of the periodontal disease led to a weight loss of approximately 8 kg, as evidenced by a decrease in BMI from 25.3 to 22.3. Improved oral hygiene may influence healthy dietary intake and weight management. Possible mechanisms include reduced systemic inflammation, better insulin sensitivity and glucose metabolism, healthier food choices, and increased overall health consciousness. Additionally, the patient reported a suppression of appetite, which might have contributed to the weight loss.

Periodontal disease affects 20%-50% of the world population [15] and is associated with cardiovascular disease, diabetes, and other systemic diseases [11]. Although the exact mechanisms of periodontitis and hepatic steatosis are not fully understood, extensive epidemiological studies have shown an association between periodontitis and the incidence of MASLD [16]. According to several cross-sectional and prospective epidemiological studies, periodontal disease is a risk factor for MASLD [4].

Several studies have investigated the role of the oral-gut-liver axis in the etiology of MASLD and periodontitis [17]. Immunogenic factors and oral pathogenic bacteria from periodontal tissue can be transmitted via hematogenous or enteral pathways and may adversely affect MASLD and MASH [17]. However, further research is needed to develop effective prevention and treatment strategies for periodontal disease against MASLD.

OLP is a chronic inflammatory disease affecting the oral mucosa, with an estimated worldwide prevalence of approximately 1% [18]. Systematic reviews and meta-analyses have shown that OLP patients have a significantly higher prevalence of liver disease, especially hepatitis C virus infection [8]. We have previously reported that the elimination of hepatitis C virus with antiviral therapy can cure OLP lesions [19] and reduce the number of periodontal pathogens (*Porphyromonas gingivalis*, *Tannerella forsythia*, *Treponema denticola*, and *Fusobacterium nucleatum*) [20].

In Japan, specialized medical institutions are responsible for treating periodontal disease, OLP, cardiac disease, and liver disease, and patients typically consult with their respective specialists in these institutions. Therefore, the associations between these different diseases are rarely evaluated comprehensively. However, the present case is unique in that the patient consulted a dental surgeon because of oral pain, and the association with liver disease was discovered during a thorough examination of the OLP and the treatment of the periodontal disease. Patients with hepatic steatosis are rarely diagnosed and treated on time due to the absence of symptoms. In this study, periodontal treatment improved the OLP lesions and liver function values, highlighting the interaction between oral health and systemic status. Long-term periodontal treatment may further improve hepatic steatosis in this patient.

## Conclusions

The findings of this study suggest that collaborations between different practice areas can significantly impact the patient’s health and must be considered in future healthcare systems and during patient education. Collaborative treatment efforts between dentistry and medicine are needed to improve the outcomes for patients with liver disease. This case report highlights the significant impact that interdisciplinary collaboration between dentistry and medicine can have on patient health. The improvement in OLP lesions and liver function values following periodontal treatment suggests a potential link between oral health and systemic diseases such as MASLD. It underscores the importance of comprehensive patient care and timely diagnosis and treatment of periodontal disease to improve overall health outcomes. Future healthcare systems should integrate collaborative approaches to better manage and treat patients with interconnected health issues.
